# Supplementary material for: Observation of vortex-antivortex pairing in decaying 2D turbulence of a superfluid gas
Source: Sci Rep. 2017 Jul 4;7:4587. doi: 10.1038/s41598-017-04122-9 (PMC5496920; doi:10.1038/s41598-017-04122-9)
Supplement: Supplementary file 1 — Supplementary Information [file 41598_2017_4122_MOESM1_ESM.pdf]

Supplementary Information:
Observation of vortex-antivortex pairing in decaying 2D turbulence in a superfluid gas

Sang Won Seo,[1] Bumsuk Ko,[1,2] Joon Hyun Kim,[1,2] and Yong-il Shin[1,2]

[1]*Department of Physics and Astronomy, and Institute of Applied Physics, Seoul National University, Seoul 08826, Korea*
[2]*Center for Correlated Electron Systems, Institute for Basic Science, Seoul 08826, Korea*

Spatial distribution of scattered atoms near a vortex

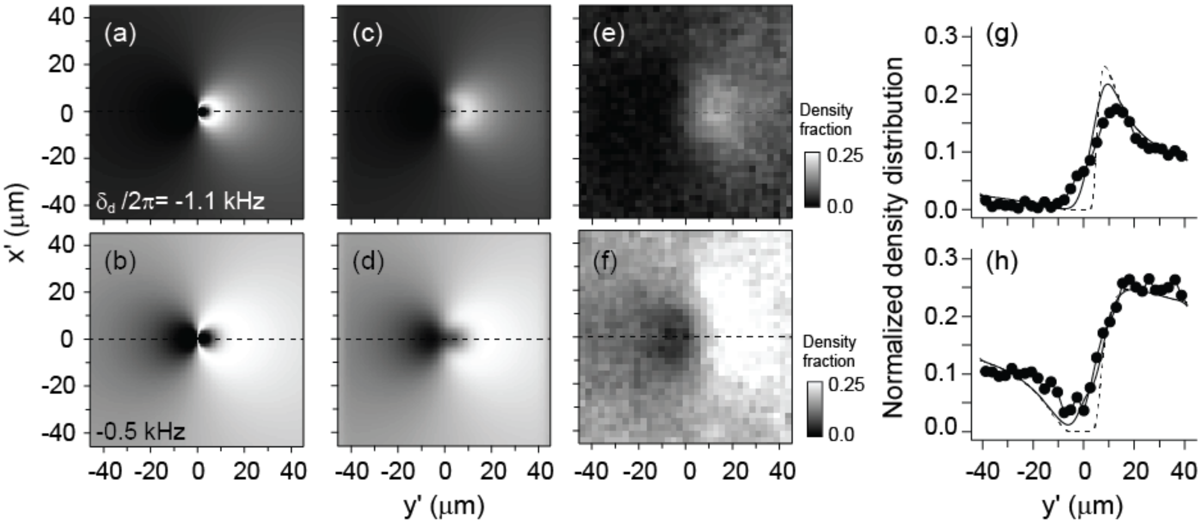

FIG. S1: Scattered atom density distribution. $\boldsymbol{n_+(x',y')/n_0}$ calculated from Eq. (1) for $\boldsymbol{S_v=-1}$, and $\boldsymbol{\frac{\delta_d}{2\pi}=-1.1}$ kHz (a) and -0.5 kHz (b). (c)-(d) Gaussian-blurred images of (a) and (b), respectively. The blurring width was set to 10 μm, which is comparable to the imaging resolution of the experimental data of $\boldsymbol{n_+(x',y')/n_0}$ for $\boldsymbol{\frac{\delta_d}{2\pi}=-1.1}$ kHz (e) and -0.5 kHz (f), obtained by averaging over ten image datasets of the vortex region cropped from scattered atom cloud images. To minimize the effect from other vortices, we used only image data where vortices are separated by over 100 μm. $\boldsymbol{n_0}$ was determined based on a Thomas-Fermi profile fit to the total atom density distribution of the initial condensate. (g)-(h) Density profile along the $\mathbf{y'}$-axis indicated by the horizontal dashed lines in (a)-(f): the experimental data (solid circles), and the theoretical calculation results with and without blurring (solid and dashed lines, respectively).

At a position of $\vec{r}$ from a vortex with sign $S_v=\pm 1$, the atom velocity is $\vec{v}(\vec{r})=s_v\hbar/(mr^2)(\hat{z}\times\vec{r})$, and for resonant Bragg scattering the frequency difference $\delta_v(\vec{r})$ of the laser beams is given by $\hbar\delta_d(\vec{r})=\frac{q^2}{2m}+\vec{q}\cdot\vec{v}(\vec{r})$, with $\hbar\delta_0=q^2/(2m)$ and $\vec{q}=\pm\sqrt{2\hbar\delta/m}(y'/r^2)$. Taking into account the spectral broadening of the Bragg scattering, which is measured using a stationary BEC in Fig. 2(c), we estimate the spatial distribution of the scattered atoms as

$$n_\pm=n_c(\vec{r})A\exp\left[-\frac{\left(\delta-\delta_v(\vec{r})\right)^2}{\delta_w^2}\right]=n_0\frac{Ar^2}{r^2+r_c^2}\exp\left[-\frac{\left(\delta_d\pm s_v\sqrt{2\hbar\delta_0/m}(y'/r^2)\right)^2}{\delta_w^2}\right], \quad (1)$$

where $n_c(\vec{r})=n_0r^2/(r^2+r_c^2)$ is the initial density distribution of the condensate. In our experiment, $A=0.25$ and $\delta_w/2\pi=615$ Hz. We set $r_c=\sqrt{2}\xi'$, where ξ' is the healing length of the condensate after a TOF of 300 μs. In Fig. S1, we compare the experimental data with the theoretical estimation and find them to be in good quantitative agreement, including possible blurring effects due to the finite imaging resolution and the expansion during the TOF.

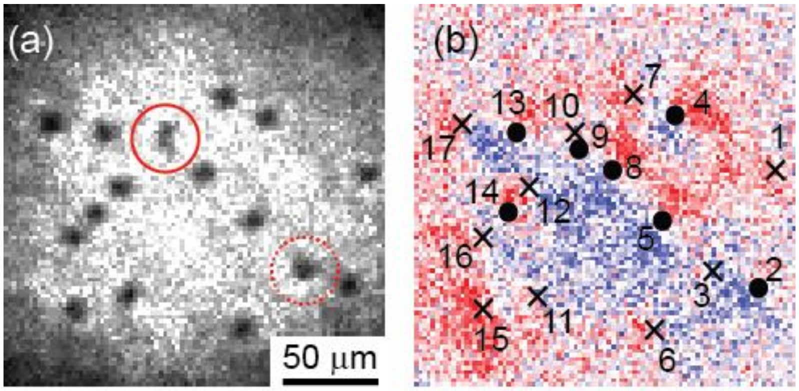

FIG. S2: Vortex sign determination. (a) Sample image of a turbulent BEC and (b) the corresponding Bragg signal $\boldsymbol{S}_{\mathbf{B}}(\boldsymbol{x}', \boldsymbol{y}')$. For most of the vortices, their signs are unambiguously determined based on the signs of the quantity $\int_{-a}^{a} \boldsymbol{sgn}(\boldsymbol{y}')\boldsymbol{S}_{\boldsymbol{B}}(\boldsymbol{x}'_i, \boldsymbol{y}'_i + \boldsymbol{y}')\boldsymbol{dy}'$, as described in the main text. However, in the case of vortex 3 (red dashed circle) and those of vortex 9 and 10 (red solid circle), the Bragg signal around the vortices is too weak to immediately determine the signs of the vortices. To vortex 3, we assign a negative sign by examining the vortex configuration of its surroundings. If vortex 3 is positive, the Bragg signal around vortices 2 and 3 should have a similar pattern to that around vortices 5 and 8. Additionally, we note that the faint blue-colored (positive $\boldsymbol{S}_{\boldsymbol{B}}$) region between vortices 2 and 3 is not compatible with vortex 3 being positive. Vortices 9 and 10 form a tight vortex dipole, showing a crescent-shaped density-depleted core region [1]. The curvature of the crescent shape indicates that the vortices are moving to the left; thus, the signs of vortices 9 and 10 are positive and negative, respectively.

Incompressible kinetic energy spectrum

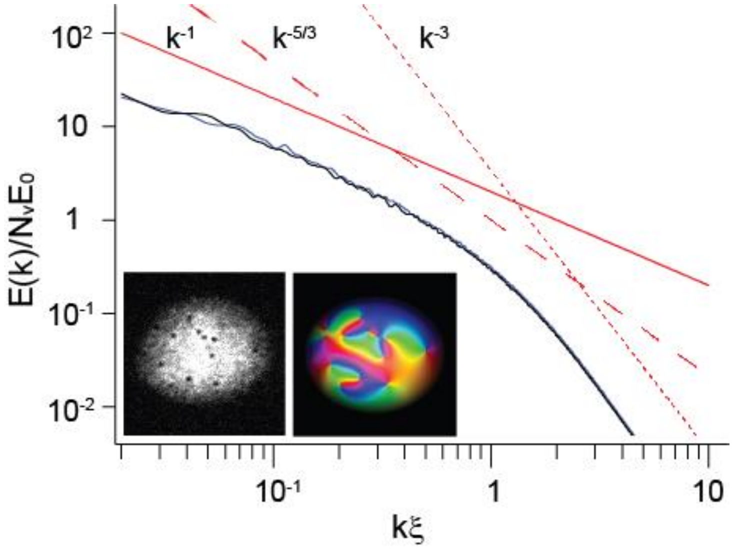

FIG. S3: Incompressible kinetic energy spectrum $E(k)$: $E(k)$ is calculated from the measured vortex configuration using Eq. (2). The blue and black lines are the averaged spectra of the experimental data for $t = 3$ s and 9 s, respectively. The insets display an image of the condensate at $t = 9$ s (left) and its phase distribution(right), which was reconstructed based on the vortex configuration information.

The incompressible kinetic energy spectrum $E(k)$ as a function of wavenumber k is the essential measure of a turbulence state, and the energy flow direction and large structure formation can be reflected in its evolution [2]. Bradley et al. [3] presented the analytic expression of $E(k)$ for a given vortex configuration $\{\vec{r}_i, s_i\}$ as

$$E(k) = N_v E_0 F(sk\xi)\left[1 + \frac{2}{N_v}\sum_{i=1}^{N_v-1}\sum_{j=i+1}^{N_v} s_i s_j J_0\left(k\left|\vec{r}_i - \vec{r}_j\right|\right)\right], \tag{2}$$

where $E_0 = sh^2\bar{n}\xi/2\pi m$ with $s =$ 1.25 and $\bar{n}$ being the column density, and $F(z) = z/4[I_1(z/2)K_0(z/2) - I_0(z/2)K_1(z/2)]^2$. J_0 is the zeroth-order Bessel function, and I_i and K_i are the ith-order modified Bessel functions of the first and second kinds, respectively. Fig. S3 shows the $E(k)$ value obtained based on our experimental data using Eq. (2). $E(k)$ exhibits k^{-3} scaling for $k > 1/\xi$, which corresponds to the vortex core structure [3]; it smoothly changes to k^{-1} scaling in the range of $2\pi/\bar{R} < k < 1/\xi$, which is the characteristic behavior for a random vortex configuration due to the $1/r$ velocity field around a vortex [3]. In our measurements there is no indication of an inertial region with $k^{-5/3}$ scaling.

[1] W. J. Kwon, G. Moon, J. Choi, S. W. Seo, and Y. Shin, Relaxation of superfluid turbulence in highly oblate Bose-Einstein condensates, Phys. Rev. A **90**, 063627 (2014).

[2] T. P. Billam, M. T. Reeves, and A. S. Bradley, Spectral energy transport in two-dimensional quantum vortex dynamics, Phys. Rev. A **91**, 023615 (2015).

[3] A. S. Bradley, and B. P. Anderson, Energy Spectra of Vortex Distribution in Two-Dimensional Quantum Turbulence, Phys. Rev. X **2**, 041001 (2012).
